# Sporadic urban leptospirosis

**DOI:** 10.3402/jchimp.v1i1.7042

**Published:** 2011-05-09

**Authors:** Elena Forouhar, Dimitra Mitsani

**Affiliations:** Union Memorial Hospital, Baltimore, MD, USA

**Keywords:** Leptospirosis, Weil Syndrome, urban, Baltimore rats

## Abstract

Severe leptospirosis (Weil Syndrome) was diagnosed in an otherwise healthy environmental worker in Baltimore alleys in late November 2010. He developed multiple organ failure but responded to antibiotic therapy and experienced a full recovery within 4 weeks. His diagnosis was confirmed by a rise in indirect hemagglutinin titer (acute 0, convalescent 400). The subject had close contact with Baltimore alley rats; a similar epidemiologic exposure and location reported in an outbreak 15 years ago.

A 33-year-old African American male with no significant past medical history presented to an emergency department in Baltimore, Maryland in late November 2010 complaining of 4 days of progressive generalized muscle pain, fever, and shaking chills. He also reported abdominal pain, diarrhea, tea-colored urine, jaundice, decreased urinary output, headache, and drowsiness. He denied smoking, alcohol, or illicit drugs. He was sexually active with one female partner. Hepatitis and HIV panel had tested negative a few months before. He denied sick contacts, recent travel, or exposure to pets, birds, new drugs, or toxins but admitted that he was a trash collector and rat trapper in streets and alleys in Baltimore and that this exposed him to a wide variety of hazards. However, he did not recall any specific recent injury such as a needle stick, animal bite, or skin laceration.

His temperature was 39.3, blood pressure was 102/67 mm Hg, pulse rate was 125 beats per minute. He was a well developed, well nourished, but ill looking African American male with rigors. He was drowsy but arousable. Sclera were icteric. Conjunctival suffusion was present. No nuchal rigidity was noted. Lungs were clear. Heart was tachycardic without murmurs. Abdomen was mildly tender in right upper quadrant without organomegaly. Both arm and calf muscles were tender to touch, pressure, and pinch. No rash was seen on the skin but a small necrotic eschar was noted on his left shin (Fig. 1). Neurologically he had no focal findings.

**Fig. 1:**
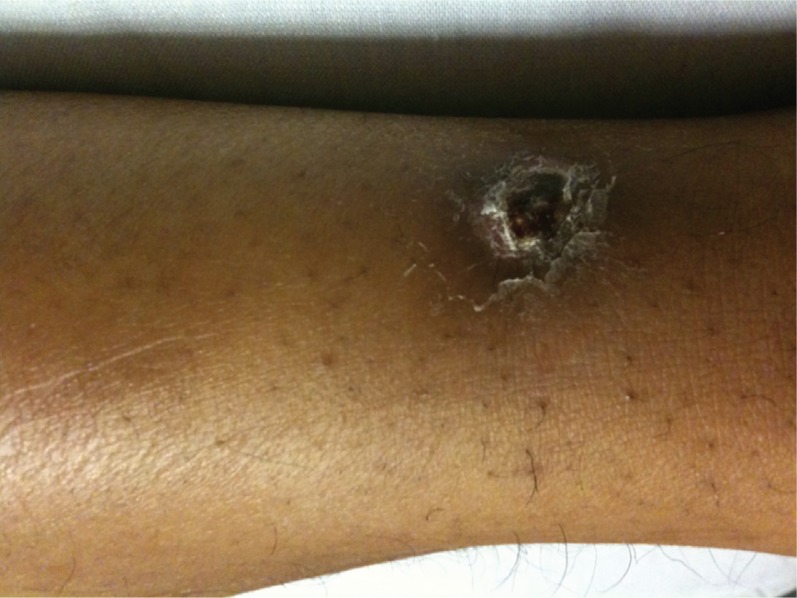
The necrotic eschar on the patient's left shin.

Significant initial lab values were: WBC: 10.3 K/UL, PMN 85.8%, and bands 13%; HgB: 12.1 g/dL; platelet count: 90 K/UL; BUN: 49 mg/dL; creatinine 4.23 mg/dL; Alkp: 134 u/L; AST: 207 u/L; ALT: 81 u/L; total bilirubin: 10.1 mg/dL with direct bilirubin 9.6 mg/dL. The CPK was 4,290 u/L, amylase 210 u/L, and lipase 2,323 u/L. No schistocytes were seen in the peripheral blood smear. Autoimmune hepatitis panel was negative. Initial microbiology included blood and urine cultures, viral hepatitis panel, HIV serology, serology for leptospira and rickettsial pox.

The patient was stabilized and started on empiric antibiotic therapy with pipercillin/tazobactam and doxycycline with suspicion for leptospirosis or other ricketsial diseases. In the first 48 hours, he became afebrile but remained aneuric. He was started on renal replacement therapy on day 4. All cultures and serology tests came back negative but serum creatinine, liver function tests, bilirubin, and lipase remained elevated despite the patient's clinical improvement (Fig. 2). Renal ultrasound, liver ultrasound, abdominal CT, MRI, and MRCP were all normal. Renal biopsy showed acute interstitial nephritis and tubular necrosis (Fig. 3). The abnormal labs peaked in the third week and returned to baseline by the fourth week.

**Fig. 2:**
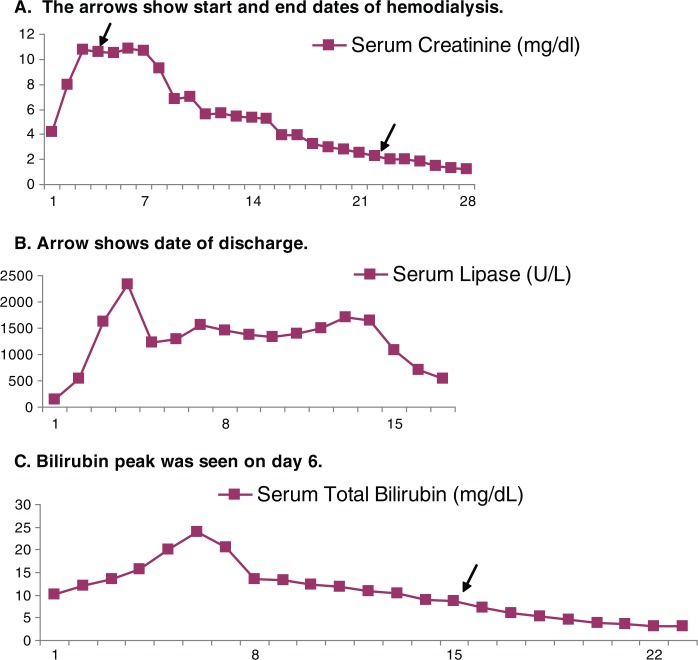
Shows the changes in patient's serum creatinine (A), serum lipase (B) and total bilirubin (C) over the course of hospitalization.

**Fig. 3:**
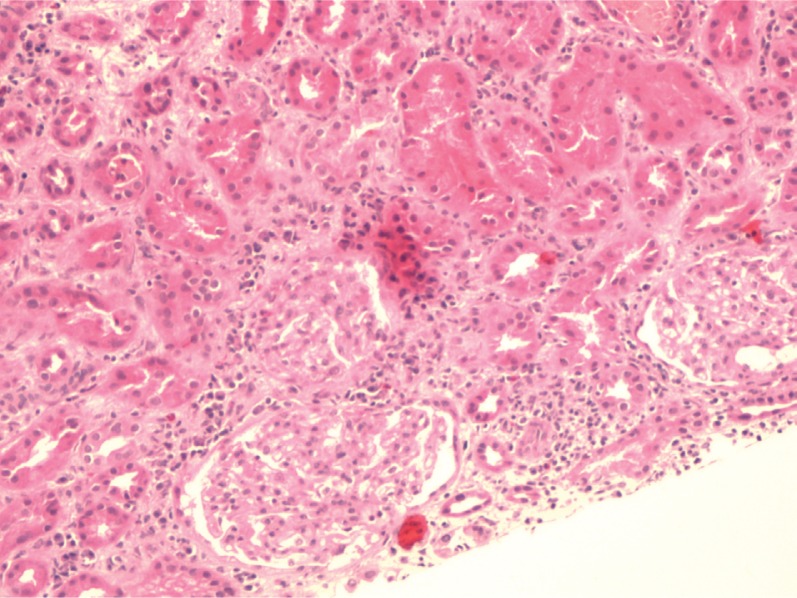
Renal Biopsy showing acute interstitial nephritis with multiple clusters of eosinophils and acute tubular injury.

Due to high clinical suspicion for leptospirosis, serology testing by indirect hemagglutination was repeated 10 days after the first sample. This was positive at 1:400 titer, confirmatory for the diagnosis.

He completed the 14-day course of antibiotic treatment. He remained on hemodialysis for a total of 16 days. In the first outpatient visit within a week after discharge, his serum creatinine was at baseline off dialysis. He experienced a full recovery.

## Discussion

Leptospirosis is a worldwide zoonotic disease caused by Leptospira species. Leptospirosis has a broad spectrum of clinical manifestations varying from unapparent infection to fulminant, fatal disease. Diagnosis is largely based upon clinical index of suspicion, and in 10–15% it presents as a severe illness that is referred to as Weil Syndrome. It is characterized by jaundice, renal dysfunction, thrombocytopenia, and hemorrhagic diathesis (1). Rodents, especially rats, are the most important reservoir although wild mammals as well as domestic and farm animals may also harbor leptospires. It is transmitted to humans following direct contact with urine, blood, or tissue from an infected animal or exposure to a contaminated environment. Water is an important vehicle (2). Leptospires build a symbiotic relationship with the host, remain in renal tubules and kidney interstitial tissue for years, and are excreted in the urine (2, 3). Certain occupational groups including veterinarians, agricultural workers, sewage workers, slaughterhouse employees, and workers in the fishing industry are at high risk. Also people involved in recreational water activities such as canoeing, windsurfing, swimming, and waterskiing are exposed to the disease (1–3). Interestingly, leptospirosis has also been recognized in inner cities and suburbs where rat populations may be expanding. Surprisingly, many inner-city residents have antibodies to *Leptospira interrogans* without having any of the above exposures (4).

An endemic substrate for the transmission of the organism is present in inner-city Baltimore. In a case series that was reported in 1996, three cases of sporadic urban leptospirosis were reported all of whom recalled walking barefoot in inner-city Baltimore alleys where they had seen rats (5). Twenty-one rats were trapped in the alleys that these patients reported as a likely location of their exposure to rat urine. Out of those, 90% were carrying *L. interrogans* in their kidney or brain detected by polymerase chain reaction (5). Two other cases have also been reported to the state between 2005 and 2009 in Baltimore County (6).

Our case was a classic presentation of Weil Syndrome who presented with acute renal failure, profound cholestasis, jaundice, thrombocytopenia, myositis, pancreatitis, and bleeding diathesis. His occupational exposure was likely to rats in alleys in Baltimore.

Rat-borne life threatening infections continue to exist in inner-city Baltimore.

## References

[1] Fauci AS, Braunwald E, Kasper DL, Hauser SL, Longo DL, Jameson JL (2008). Harrison's principles of internal medicine.

[2] Bharti AR, Nally JE, Ricaldi JN, Matthias MA, Diaz MM, Lovett MA (2003). Leptospirosis: a zoonotic disease of global importance. Lancet Infect Dis.

[3] Nardone A, Capek I, Baranton G, Campèse C, Postic D, Vaillant V (2004). Risk factors for leptospirosis in metropolitan France: results of a national case–control study, 1999–2000. Clin Infect Dis.

[4] Maroun E, Kushawaha A, El-Charabaty E, Mobarakai N, El-Sayegh S (2011). Fulminant Leptospirosis (Weil's disease) in an urban setting as an overlooked cause of multiorgan failure: a case report. J Med Case Reports.

[5] Vinetz JM, Glass GE, Flexner CE, Mueller P, Kaslow DC (1996). Sporadic urban leptospirosis. Ann Intern Med.

[6] Maryland Infectious Disease and Environmental Health Organization website Reportable diseases counts and rates. http://ideha.dhmh.maryland.gov/reportable-diseases.aspx.

